# The effect of age and gender on HbA1c levels in adults without diabetes mellitus

**DOI:** 10.5937/jomb0-44190

**Published:** 2023-10-27

**Authors:** Şener Gülsen, Kahvecioğlu Esra Deniz, Can Başak, Gümüş Alper, Beyazıt Semih Yeşil, Evran Betül

**Affiliations:** 1 Başakşehir Çam and Sakura City Hospital, Department of Biochemistry, Istanbul, Turkey; 2 Başakşehir Çam and Sakura City Hospital, Department of Internal Medicine, Istanbul, Turkey

**Keywords:** diabetes mellitus, HbA1c, gender, age, correlation, dijabetes melitus, HbA1c, pol, starost, korelacija

## Abstract

**Background:**

Hemoglobin A1c (HbA1c) levels play an important role in diagnosing, screening, and monitoring the treatment of diabetes. Our study aims to determine whether a relationship exists between HbA1c levels and age and gender in Turkish adults who have not been diagnosed with diabetes.

**Methods:**

This retrospective study included 6776 Turkish adults with no known diabetes. Cross-sectional analyses of A1C levels were performed between different age and gender categories. In statistical analysis, t-test, linear regression analysis, one-way ANOVA analysis, and LSD post hoc were used.

**Results:**

HbA1c levels in the individuals examined by dividing into different age groups increased with age in all groups. In our study, HbA1c levels were significantly higher in males than females (p<0.001) in all groups, especially between the ages of 30-49, and were positively associated with age for males and females. There was a positive correlation between HbA1c and age in both mans and women aged 30-49 (P<0.05). In the HbA1c 6.5% group newly diagnosed with diabetes, HbA1c levels gradually decreased with age in both genders, and no significant effect of age on HbA1c level was detected (p>0.05).

**Conclusions:**

Our results showed that it is important to evaluate the effects of age and gender when using HbA1c levels in the diagnosis, screening, and treatment of diabetes, especially in young and middle-aged populations. Applying this situation to daily practice may reduce the misdiagnosis of diabetes in elderly patients, overtreatment of diabetes, and its associated risks.

## Abstract (sr)

**Uvod:**

Nivoi hemoglobina A1c (HbA1c) igraju važnu ulogu u dijagnostikovanju, skriningu i praćenju tretmana dijabetesa. Na{a studija ima za cilj da utvrdi da li postoji veza između nivoa HbA1c i starosti i pola kod turskih odraslih osoba kojima nije dijagnostikovan dijabetes.

**Metode:**

Ova retrospektivna studija je obuhvatila 6776 turskih odraslih osoba bez dijabetesa. Izvršena je analiza nivoa A1C kod različitih starosnih i polnih kategorija. U statističkoj analizi su korišćeni t-test, linearna regresiona analiza, jednofaktorska ANOVA analiza i post hoc test sa LSD korekcijom.

**Rezultati:**

Nivoi HbA1c kod ispitanika su ispitivani tako {to su podeljeni u različite starosne grupe. Nivoi HbA1c su se povećavali sa rastom životne dobi u svim grupama. U našoj studiji, nivoi HbA1c su bili značajno vi{i kod muškaraca u odnosu na žene (p<0,001) u svim grupama, posebno između 30. i 49. godine života, i bili su pozitivno povezani sa starošću kod muškaraca i žena. Postojala je pozitivna korelacija između HbA1c i starosti kod muškaraca i žena u dobi od 30-49 godina (p<0,05). U grupi sa HbA1c 6,5% kojima je nedavno dijagnostikovan dijabetes, nivo HbA1c se postepeno smanjivao s godinama kod oba pola, i nije primećen značajan uticaj godina na nivo HbA1c (p>0,05).

**Zaključak:**

Naši rezultati pokazuju da je važno proceniti uticaj starosti i pola prilikom korišćenja nivoa HbA1c u dijagnostici, skriningu i tretmanu dijabetesa, posebno kod mladih i sredovečne populacije. Primena ove situacije u svakodnevnoj praksi može da smanji netačnu dijagnozu dijabetesa kod starijih pacijenata, preterano lečenje dijabetesa i povezane rizike.

## Introduction

Glycemia changes with age, and the prevalence of diabetes and prediabetes increases with ageing worldwide [1]. Considering that the elderly population constitutes almost half of all adults diagnosed with Type 2 Diabetes Mellitus (Type 2DM), it is essential to consider the effects of ageing on the glycemic level in diagnosing and managing diabetes [2].

According to the criteria of the American Diabetes Association (ADA), the diagnosis of diabetes can be made based on glucose or oral glucose tolerance test or Hemoglobin A1c (HbA1c) levels [3]. HbA1c, which is directly related to the amount of glucose, is more advantageous and widely used because it does not require fasting, can be taken at any time, is not affected by changes in diet, shows less variability during acute illness, has higher preanalytical stability and is standardised [3]. HbA1c plays a critical role in managing diabetic patients, especially as it is used as a marker to assess metabolic status, determine the risk of diabetes complications, and guide treatment [4] [5]. HbA1c gives information about the average glucose concentration of 100-120 days before the measurement date, and HbA1c levels of 6.5% for diabetes were accepted as diagnostic criteria [6] [7]. Some studies have shown that metabolic control changes in non-diabetic individuals and HbA1c levels increase with age due to the effect of the physiological process [4] [6] [8]. However, current HbA1c targets for the diagnosis and treatment of diabetes are not age-specific, and the effect of age is usually not considered [3] [9]. The HbA1c reference range used in most laboratories for all age groups was obtained from a few healthy volunteers younger than 40 in 1986 [10]. Using a single cut-off value for diabetes diagnosis by ignoring age and gender-related physiological changes for HbA1c may lead to misdiagnosis. Again, HbA1c targets, which do not take into account age in monitoring glycemia, maybe too strict for elderly diabetic patients as they lead to high hypoglycemia and drug side effects as a result of unnecessary overtreatment [11]. Therefore, considering the increase in the prevalence of Type 2 DM and the role of HbA1c in the diagnosis, treatment, and management of diabetes, it becomes more important to determine appropriate HbA1c targets by investigating the effects of age and gender-related physiological factors on HbA1c.

This study aims to determine the relationship between HbA1c levels with age and gender in a large Turkish sample that has not been diagnosed with diabetes. Thus, important clinical information can be provided to avoid unnecessary diabetes diagnosis and treatment in the elderly.

## Materials and methods

In this retrospective study, a total of 6776 Turkish adults (3088 males and 3688 females) with no known diabetes aged between 18-97 years, who applied to the Internal Medicine outpatient clinic of Ba ak ehir Çam and Sakura City Hospital between January 2021 and May 2022 were included. Individuals under the age of 18, pregnant women, those with malignancy, patients with a diagnosis of diabetes, those with renal failure, those using corticosteroids, and those with anaemia and hemoglobinopathy were not included in the study. Diabetes is defined as HbA1c 6.5% (47.5 mmol/mol) or fasting blood glucose 126 mg/dl (6.99 mmol/L) or random blood glucose 200 mg/dl (11.1 mmol/L) or oral glucose tolerance test second hour glucose 200 mg/dl (11.1 mmol/L) according to ADA criteria [3]. HbA1c 6.5% (47.5 mmol/mol) were categorised as diabetes. In addition, the data of the patient group with high HbA1c levels and newly diagnosed diabetes were also analysed according to age and gender. Age, gender, and HbA1c data were obtained from the hospital information system, analysed, and compared.

This study is in accordance with the Declaration of Helsinki. This study was approved by the ethics committee of Istanbul Başakşehir Çam and Sakura City Hospital (No: 2022.07.229). Due to the retrospective and observational character of the study design, the requirement for informed consent has been waived. 

For HbA1c measurement, venous blood samples from the patients were taken into tubes containing K2EDTA and HbA1c measurements were performed within 4 h of sampling. HbA1c levels were analysed in Istanbul Başakşehir Çam and Sakura City Hospital Central Laboratory. HbA1c was performed by multicapillary zone electrophoresis method using original reagents in Sebia Capillarys 3 Tera (Sebia/France) instrument. The Capillarys 3 instrument uses the principle of capillary electrophoresis in free solution, the most common form of capillary electrophoresis. This technique separates charged molecules by their electrophoretic mobility in an alkaline buffer with a specific pH. Separation also occurs according to the electrolyte pH and electroosmotic flow. A sample dilution with hemolysing solution is prepared and injected by aspiration at the anodic end of the capillary. A high-voltage protein separation is then performed, and the haemoglobins are directly detected at the capillary's cathodic end at 415 nm, which is the absorbance wavelength specific to haemoglobins. The inter- and intra-assay coefficients of variation were 0.96% and 0.85%, respectively, for HbA1c. 

Data for normally distributed variables were presented as mean and standard deviation (SD) and as frequency and percentage for categorical variables. In comparing the obtained HbA1c levels by gender, a ttest was used in independent groups, and linear regression analysis was used to determine the effect of age and gender on HbA1c. The variation of HbA1c measurement according to age groups was examined by one-way ANOVA analysis. In case of significant differences after a one-way analysis of variance, the LSD post hoc test was used to determine which group the difference originated from. Linear regression models were tested to determine the effect of age on HbA1c level. Analyses were performed with IBM SPSS Statistics version 20.0 software at a 95% confidence level.

## Results

A total of 6776 adults, 3088 (45.6%) male and 3688 (54.4%) female, were included in the study. The mean age of the subjects was 46.66±15.4 years, and the mean HbA1c level was 5.65±0.81%; 6.5% (n=438) of the study participants met the criteria for diabetes as they had HbA1c 6.5. In the HbA1c <6.5 and HbA1c 6.5 groups, there were differences in HbA1c levels between males and females, and HbA1c was significantly higher in males than females in both groups (p<0.001) (Table 1).

**Table 1:** Comparison of HbA1c values by gender in HbA1c<6.5 and HbA1c 6.5 groups. n=The number of participants, SD=Standart deviation, independent t-test

		HbA1c (%)			t	p
		n	Mean	SD		
HbA1c <6.5 group	Female	3529	5.45	0.37	-10.051	<0.001
	Male	2809	5.55	0.39		
HbA1c ≥6.5 group	Female	159	7.46	1.35	-3.812	<0.001
	Male	279	8.05	1.87		

Significant differences in the mean of measurements between HbA1c <6.5 group and HbA1c 6.5 groups in women and men were examined with the ttest in independent groups, and the HbA1c levels obtained separately in women and men showed significant differences (p<0.001) (Table 2). When the mean HbA1c levels of women and men were examined by t-test in independent groups, a significant difference was observed in the mean of women and men in all groups. In all groups, men had significantly higher HbA1c levels than women.

**Table 2:** Comparison of HbA1c (%) values by gender in HbA1c <6.5 and HbA1c ≥6.5 groups. n=The number of participants, SD=Standart deviation, t-test

	Total			HbA1c <6.5 group			HbA1c 6.5 group			
	n	Mean	SD	n	Mean	SD	n	Mean	SD	p
Female	3688	5.54	.61	3529	5.45	.37	159	7.46	1.35	<0.001
Male	3088	5.77	.98	2809	5.55	.39	279	8.05	1.87	<0.001
p	<0.001			<0.001			<0.001			

It was tested with linear regression models to examine the effect of age on HbA1c levels at different age levels in women and men in the study groups. While age has a positive effect on HbA1c level in women aged 30-39 years (p<0.001) and 40-49 years (p<0.001) in the HbA1c <6.5 group, age has no significant effect on HbA1c level in other age groups. Age did not have a significant effect on HbA1c levels in men aged 30-39 years (p=0.018), 40-49 years (p=0.001), and 50-59 years (=0.033) in the same group (Table 3). Age did not have a significant effect on HbA1c level for all age groups of women and men in the HbA1c 6.5 group (p>0.05) (Table 3).

**Table 3:** Linear regression of age and HbA1c. β= Beta value, SE= Standard error

HbA1c <6.5%	Male	β	SE	t	p
	All age	0.524	0.0004	36.552	<0.001
	18–29	-0.018	0.0041	-0.427	0.669
	30–39	0.142	0.0036	4.049	<0.001
	40–49	0.207	0.0036	6.518	<0.001
	50–59	0.075	0.0044	1.913	0.056
	60–69	0.11	0.0055	1.985	0.048
	>=70	-0.02	0.0034	-0.294	0.769
HbA1c <6.5%	Male				
	All age	0.434	0.0004	25.537	<0.001
	18–29	0.035	0.0047	0.698	0.485
	30–39	0.107	0.0052	2.367	0.018
	40–49	0.124	0.0049	3.24	0.001
	50–59	0.086	0.0048	2.143	0.033
	60–69	0.006	0.0070	0.104	0.917
	>=70	0.085	0.0038	1.419	0.157
HbA1c ≥6.5%	Female				
	All age	-0.184	0.0078	-2.38	0.019
	18–29	-	-		-
	30–39	-0.266	0.2371	-0.826	0.430
	40–49	-0.219	0.0821	-1.384	0.174
	50–59	0.127	0.0922	0.749	0.459
	60–69	-0.237	0.0746	-1.467	0.151
	>=70	-0.072	0.0320	-0.400	0.692
HbA1c ≥6.5%	Male				
	All age	-0.222	0.0091	-3.788	<0.001
	18–29	0.286	0.3052	0.422	0.714
	30–39	-0.156	0.2143	-0.546	0.595
	40–49	0.088	0.1041	0.748	0.457
	50–59	0.095	0.0651	0.884	0.379
	60–69	0.033	0.0669	0.255	0.799
	>=70	-0.057	0.0405	-0.341	0.735

**Figure 1:**
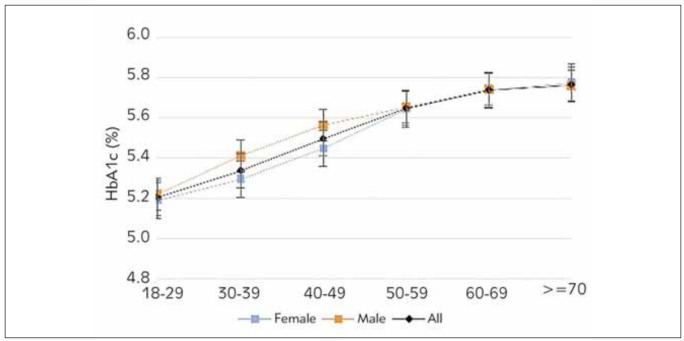
HbA1c concentrations for different age groups in the HbA1c <6.5 group.

**Figure 2:**
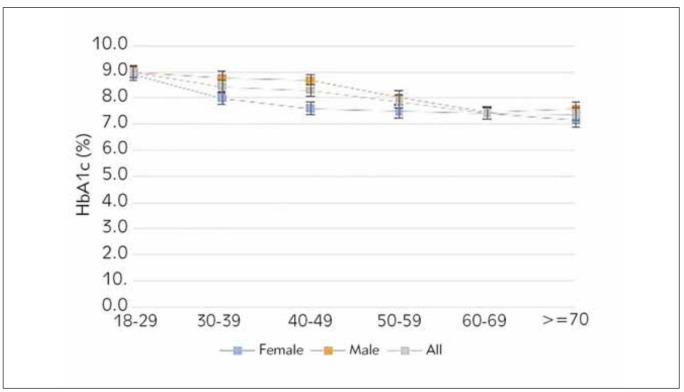
HbA1c concentrations for different age groups in the HbA1c ≥6.5 group.

In HbA1c <6.5 group and HbA1c 6.5 groups, HbA1c levels of women and men in different age groups were examined by t-test in independent groups. Accordingly, HbA1c <6.5; While there was a significant difference between women and men in the 30-39 and 40-49 age groups (p<0.05), there was no significant difference between women and men in the other age groups. For the 30-39 and 40-49 age groups, which showed significant differences, men had significantly higher Hb1Ac levels than women (Table 4) (Figure 1). In the HbA1c 6.5% group, HbA1c levels gradually decreased with age in both sexes. While there was a significant difference between women and men in the 40-49 age group (p=0.009), there was no significant difference between women and men in other age groups (Table 4) (Figure 2).

**Table 4:** HbA1c (%) levels of women and men in different age groups in HbA1c <6.5 and HbA1c ≥6.5 groups. n=The number of participants, SD=Standart deviation, t–test

		Total			Female			Male			p
		n	Mean	SD	n	Mean	SD	n	Mean	SD	
HbA1c <6.5%	18–29	989	5.20	0.30	589	5.19	0.28	400	5.22	0.32	0.111
	30–39	1275	5.34	0.30	793	5.29	0.28	482	5.41	0.32	<0.001
	40–49	1620	5.49	0.35	947	5.44	0.33	673	5.56	0.36	<0.001
	50–59	1278	5.65	0.33	655	5.64	0.33	623	5.65	0.33	0.72
	60–69	676	5.74	0.35	323	5.74	0.32	353	5.74	0.37	0.916
	>=70	500	5.76	0.35	222	5.77	0.32	278	5.76	0.37	0.537
	p	<0.001			<0.001			<0.001			
HbA1c ≥6.5%	18–29	5	8.98	1.57	1	8.90		4	9.00	1.82	0.964
	30–39	25	8.42	1.85	11	7.99	1.70	14	8.76	1.95	0.310
	40–49	113	8.28	2.11	40	7.59	1.45	73	8.66	2.32	0.009
	50–59	124	7.86	1.63	36	7.47	1.41	88	8.02	1.69	0.089
	60–69	100	7.42	1.34	38	7.40	1.25	62	7.43	1.40	0.905
	>=70	71	7.37	1.32	33	7.13	1.08	38	7.57	1.49	0.161
	p	<0.001			0.386			<0.001			

In HbA1c <6.5 group, in the whole study group, women's and men's Hb1Ac levels differ significantly according to age groups (p<0.05). According to the results of the TUKEY test performed to determine which group the difference originates from, there is no significant difference between 60-69 and 70 years of age in women and men, and Hb1Ac levels increase as the age group increases.

In HbA1c 6.5 group, when the change of HbA1c level in the whole study group, women and men according to age groups was examined; while it was observed that HbA1c levels differed significantly according to age groups in the men and entire study group (p<0.05), it was not found to differ significantly in women. According to the results of the TUKEY test, which was carried out to determine which group the difference originated from for the men and the entire study group with a significant difference, in all of the HbA1c 6.5 group and men, people aged 40-49 have significantly higher HbA1c levels than those aged 60-69 and 70.

## Discussion

HbA1c is an important marker used for the diagnosis and follow-up of diabetes. Nevertheless, using a single cut-off value for diabetes diagnosis by ignoring age and gender-related physiological changes for HbA1c may lead to misdiagnosis. In a study of 4748 people, the effect of age and gender on HbA1c levels for non-diabetic Taiwanese adults was examined and found to be effective [12]. However, these relationships have not been adequately explored for Turkish adults without diabetes. We examined whether HbA1c changes with age and gender in a large, nondiabetic sample of Turks. Our study found that HbA1c levels were significantly higher in males than females, increasing with age, and HbA1c was positively associated with age for both males and females.

In their study of non-diabetic individuals, Roth et al. reported that HbA1c levels gradually increase with age, resulting in a 0.074% increase in HbA1c per decade [8]. The results of our study showed that HbA1c levels were positively correlated with age, as reported in various previous studies [13] [14] [15]. It has been stated that glucose and HbA1c levels increase as a result of deterioration in pancreatic -cell function, decrease in insulin receptor activity, insulin sensitivity, muscle mass, and glucose consumption with increasing age [16].

Hashimoto et al. found an increase in HbA1c with age in those without a family history of diabetes. They stated that glycohemoglobin levels were affected by age independently of the diabetes trends [13]. Various studies have suggested that the increase in HbA1c levels may be independent of glucose metabolism disorder [15] [17]. This shows that the increase in glycation, which is caused by ageing and is one of the causes of diabetic complications, is not only dependent on blood glucose concentration [8] [17]. Therefore, it has been reported by Pani et al. that the upper limit of HbA1c can be considered up to 6.60% in the elderly without glucose homeostasis abnormalities [17].

Changes also in erythrocyte (RBC) turnover or clearance may contribute to the increase in HbA1c with together age, independent of impaired metabolic control [15] [18]. Wu et al. found that the number of red blood cells decreased with age [18]. A decrease in the number of RBCs can lead to a prolongation of RBC lifespan and an increase in HbA1c levels. It has been suggested that an increase in HbA1c may occur as a result of prolonged glucose exposure caused by decreased clearance due to impaired macrophage function [17] [18]. It has been stated that cellular damage resulting from decreased membrane lipids, enzyme activity and increased cell fragility in erythrocytes as a result of ageing may also accelerate glycation [17] [18]. It has also been reported that besides the renal dysfunction that develops with ageing, B12 and iron deficiency and impaired spleen function are associated with increased HbA1c levels independent of hyperglycemia [15]. Therefore, the HbA1c level may not fully reflect the mean glucose levels in elderly individuals [17].

In addition, our study showed no significant difference between HbA1c and age in men and women aged 50-70 years. Similar results were also obtained for men in a study conducted in Taiwanese [12]. This difference may be due to lifestyle changes. Despite the deterioration in glucose tolerance, how the decrease in the age-related increase pattern occurs has not been fully elucidated. However, it has been stated that it may be caused by inadequate dietary intake with nutritional changes and ageing [19].

Our data showed that HbA1c levels are higher in men than in women and increase with age, similar to various studies, so it is important to consider age and gender in the use of HbA1c [12] [16] [20]. Hovestadt et al. In their study of 2455 healthy children and adolescents, they found that HbA1c was higher in males and increased with age and stated that there was a post-childhood gender difference in healthy individuals [4].

A study conducted with 135,893 people showed that HbA1c level was higher in men than in women in the 20-59 age group [6]. In the study of Huang et al., [12] similar to our study, HbA1c levels were found to be significantly higher in men in the 30-49 age group than in women in the same age group [12]. Cohen et al. [21] showed that the gender-based difference in RBC lifespan significantly affected HbA1c values in individuals. One of the reasons for this has been shown to be a shorter RBC survival time resulting in shorter glucose exposure [22]. During menstruation, rapid erythrocyte turnover is associated with lower haemoglobin levels, which may result in lower HbA1c levels than men [16] [19]. This situation may explain women's significantly lower HbA1c levels, especially in the 30-49 age group, as in adults from China and Taiwan [12] [16]. Estrogen has been reported to play a role in suppressing erythropoiesis both in vitro and in vivo [14] [23]. In addition, estrogen positively affects glucose metabolism as a result of an increase in insulin secretion and sensitivity [24]. Similar to the study of Simon et al. [25], we found that although HbA1c levels in women increase with age, there is no gender difference after age 50. In a conducted study, the age of menopause in Turkey was found to be 50.8 [26]. This may explain the decreased HbA1c difference between sexes after 50 years of age in Turkish adults.

The large sample size, age distribution range, and random selection of our study are strengths. However, our study has some limitations. Although our database provided a large sample size and reliable data, there were differences in the age and gender distribution of the groups. In our study, HbA1c <6.5% was our only criterion for classifying the definition of patients without diabetes. Therefore patients with undetected impaired glucose tolerance or diabetes mellitus may be found, although the effect is probably not significant since a glucose tolerance test could not be performed. Since the study was conducted in a single centre, it does not reflect the entire Turkish population. However, it is a source for studies on the relationship of HbA1c with age and gender. We think prospective studies with a wider age range of participants, detecting impaired fasting glucose and/or impaired glucose tolerance, need to elucidate the relationship between HbA1c levels and age and the causal mechanisms.

Our study investigated the relationship of HbA1c levels with age and gender in Turkish adults in a non-diabetic, relatively large sample. Our data showed that age was a factor in causing increased HbA1c levels. HbA1c levels were lower in women than men, with more pronounced in premenopausal periods. In summary, we found that age and gender differences affect HbA1c concentrations. Increased HbA1c levels suggest the role of nonglycemic factors. Although the prevalence of prediabetes is high in advanced-age adults, progression to diabetes is not common. However, with advanced age, both hypoglycemia symptoms and responses are reduced, and an increase in hypoglycemia is sometimes seen due to excessive glycemic control. Therefore, in diagnosing and treating diabetes and prediabetes, determining appropriate HbA1c targets by considering age and gender-related differences, developing the right strategy, preventing misdiagnosis, intensive treatment, hypoglycemia, and drug side effects will be extremely beneficial. We think that the question of whether a cut-off is required for HbA1c according to age and gender, rather than a single reference range for all ages and genders, should be addressed in a clinical study and examined in more detail.

## Dodatak

### Ethical approval

This study was approved by the ethics committee of Istanbul Başakşehir Çam and Sakura City Hospital (No:2022.07.229).

### Conflict of interest statement

All the authors declare that they have no conflict of interest in this work.
